# TNF up-regulates Pentraxin3 expression in human airway smooth muscle cells via JNK and ERK1/2 MAPK pathways

**DOI:** 10.1186/s13223-015-0104-y

**Published:** 2015-12-07

**Authors:** Jingbo Zhang, Latifa Koussih, Lianyu Shan, Andrew J. Halayko, Ben-Kuen Chen, Abdelilah S. Gounni

**Affiliations:** Department of Immunology, Faculty of Health Sciences, College of Medicine, University of Manitoba, Winnipeg, MB Canada; Physiology and Physiopathology, Faculty of Health Sciences, College of Medicine, University of Manitoba, Winnipeg, MB Canada; Institute of Bioinformatics and Biosignal Transduction, College of Bioscience and Biotechnology, National Cheng Kung University, Tainan, Taiwan; Department of Nephrology, Xinqiao Hospital, Third Military Medical University, Chongqing, 400037 China; Universite de Saint Boniface, 200 Avenue de la cathedrale, Winnipeg, MB Canada

**Keywords:** Pentraxin-3, TNF, Airway smooth muscle cells, Signaling

## Abstract

**Background:**

Long pentraxin 3 (PTX3) is a novel candidate marker for inflammation in many chronic diseases. As a soluble pattern recognition receptor, PTX3 is involved in amplification of inflammatory reactions and regulation of innate immunity. Previously, we demonstrate that human airway smooth muscle cells (HASMC) express constitutively PTX3 and upon TNF stimulation. However, very little is known about the mechanism governing its expression in HASMC. We sought to investigate the mechanism governing TNF induced PTX3 expression in primary HASMC.

**Methods:**

HASMC were stimulated with TNF in the presence of transcriptional inhibitor actinomycin D (ActD) or MAPKs pharmacological inhibitors. PTX3 mRNA and protein expression were analyzed by Real-time RT-PCR and ELISA, respectively. PTX3 promoter activity was determined using luciferase assay.

**Results:**

PTX3 mRNA and protein are expressed constitutively by HASMC and significantly up-regulated by TNF. TNF-induced PTX3 mRNA and protein release in HASMC were inhibited by transcriptional inhibitor actinomycin D. TNF induced significantly PTX3 promoter activation in HASMC. MAPK JNK and ERK1/2 specific inhibitors (SP600125 and UO126), but not p38, significantly down regulates TNF induced PTX3 promoter activity and protein release in HASMC. Finally, TNF mediated PTX3 promoter activity in HASMC was abolished upon mutation of NF-κβ and AP1 binding sites.

**Conclusions:**

Our data suggest that TNF induced PTX3 in HASMC at least via a transcriptional mechanism that involved MAPK (JNK and ERK1/2), NF-κβ and AP1 pathways. These results rise the possibility that HASMC derived PTX3 may participate in immune regulation in the airways.

## Background

Asthma is a major cause of morbidity and mortality worldwide. It is a chronic inflammatory condition of the airways characterized by bronchial hyperresponsiveness, infiltration of inflammatory cells, and airway remodeling [1].

Airway smooth muscle cells (ASMC) are one of the tissue-forming lung cells that have recently gained appreciation as one of the major contributors to asthma pathogenesis [2]. It is now established that the ASMC are rich source of different pro-inflammatory factors, such as cytokines, chemokines, and respond to their action via modulating key cellular functions such as cell proliferation, migration and inflammatory cell interaction culminating in airway remodeling [3].

Tumor necrosis factor (TNF) is a well characterized proinflammatory cytokine that plays a central role in asthma pathogenesis through direct immunomodulatory actions on ASMC [4]. TNF alone or in combination with other cytokines enhances the expression of various mediators including cytokines, chemokines adhesion molecules among others [2, 4]. Notably, TNF is one of the most potent activators of MAPK, AP1 and NF-κB signaling pathways leading to upregulation of cellular genes involved in immune inflammatory processes [5].

Pentraxin 3 (PTX3), the prototype long pentraxin, is an acute phase protein which can be produced by a variety of cells at the site of infection or inflammation [6]. Unlike short pentraxins (e.g. CRP) which are induced in liver by IL-6, PTX3 expression is induced predominantly by proinflammatory cytokines such as IL-1β but not IL-6. The PTX3 levels are very low in serum and tissue compartments of normal subjects but are rapidly increased in response to inflammation triggered by infections, autoimmunity, or mechanical stress (reviewed in [7]). Cumulative evidence suggests that the PTX3 could serve as a useful new serological marker, rapidly reflecting tissue inflammation and damage under diverse clinical conditions [8].

In context of lung, despite a reported protective effect of PTX3 in infection by certain fungi, bacteria, or viruses, enhanced expression of PTX3 was associated with more severe lung injury. Interestingly, PTX3 is widely accepted as a marker of severity and an outcome predictor in acute lung injury (ALI)/acute respiratory distress syndrome (ARDS) patients [7, 9]. The role of PTX3 in airway obstructive diseases remains yet to be clarified. Although in COPD patients, PTX3 level correlates with forced expiratory volume in 1 s (FEV1), and enhanced PTX3 is found in induced sputum of COPD patients compared to healthy controls; multiple studies have failed to establish a definitive role [10–12]. More recently, Doni et al. have demonstrated that the absence of PTX3 leads to tissue damage exacerbation in mouse model of skin inflammation [13]. Furthermore, an essential role of PTX3 derived mesenchymal stromal cells was revealed in wound repair suggesting a plausible role of PTX3 in regulating airway remodeling [14]. In allergic asthma, we have shown that PTX3 is highly expressed within the airway of allergic asthmatics compared with healthy donors [15]. HASMC, and to lesser extent epithelial cells, are the main producers of PTX3 in vitro at the baseline and upon TNF stimulation [15]. However, the mechanism governing TNF induction of PTX3 in ASM cells is not fully understood. Therefore, the aim of this study was to investigate the mechanism by which TNF regulates this molecule in HASMC.

## Methods

### Reagents

Recombinant human TNF, PTX3 protein, and ELISA kit for human PTX3 were purchased from R&D Systems (Minneapolis, MN). The p38 mitogen-activated protein kinase (MAPK) inhibitor, SB-203580 (4-[4-fluorophenyl]-5-[4-pyridyl] 1Himidazole); the MEK-1/2 extracellular signal-regulated kinase p42/p44 ERK inhibitor, U-0126 (1,4-diamino-2,3-dicyano-1,4-bis[2-aminophenyl-thio]butadiene); and the c-jun N-terminal kinase JNK inhibitor, SP600125 (1,9-Pyrazoloanthrone) were purchased from Calbiochem (Mississauga, ON, Canada). DMEM, Ham’s F12, trypsin–EDTA, antibiotics (penicillin, streptomycin), were from Invitrogen Life Technologies (Grand Island, NY). All other reagents were procured from Sigma-Aldrich Canada Ltd. (Oakville, ON, Canada), unless specified.

### HASMC preparation and cell culture

This study was approved by the Human Research Ethics Board of the University of Manitoba, Winnipeg, Canada. Primary human ASM cells (HASMC) of tracheal origin were obtained from macroscopically healthy segments of the trachea after lung resection from surgical patients in accordance with procedures approved by the Human Research Ethics Board of the University of Manitoba, Winnipeg, Canada. Primary bronchial HASMC were isolated from explants as described previously [16]. A written consent was obtained from all the subjects involved in this study.

At confluence, primary HASMC exhibited spindle morphology and a hill-and-valley pattern that is characteristic of smooth muscle in culture. Furthermore, the cells at confluence retain smooth muscle-specific actin, SM22, and calponin protein expression and mobilize intracellular Ca^2+^ in response to acetylcholine, a physiologically relevant contractile agonist [16].

To test the effect of TNF on PTX3 mRNA and protein expression, cells were cultured on uncoated plastic dishes in Dulbecco’s modified Eagle medium complete medium (DMEM supplemented with l-glutamine (2 mM), 100 μg/ml streptomycin, 100 U/ml penicillin, and 10 % fetal bovine serum) at 37 °C with 5 % CO_2_. Unless otherwise indicated, cells were cultured to 70 % sub-confluency and serum deprived for 48 h in Ham’s F12 medium supplemented with 100 μg/ml streptomycin, 100 U/ml penicillin, and 1XITS (5 μg/ml insulin, 5μg/ml transferring and 5 ng/ml selenium) before each experiment. Serum deprived HASMC were then stimulated in fresh FBS-free F-12 medium with human TNF (10 ng/ml), or medium alone at indicated time. For pharmacological inhibition studies, cells were pretreated for 1 h with U0126 (10 µM), SB203580 (10 µM), and SP600125 (50 nM) before stimulation for 24 h with TNF (10 ng/ml). Supernatants were collected, cleared by centrifugation and stored in −80 °C until analyzed by ELISA. In all experiments, cells were used at passages 3–5.

### ELISA analysis of protein release in cell supernatants

PTX3 was quantified using ELISA according to protocol provided by the manufacturer (R&D systems) as we previously described [17]. ELISA sensitivity for PTX3 was 20 pg/ml.

### Quantitative real-time RT-PCR analysis

Serum deprived HASMC were treated with TNF (10 ng/ml) or vehicle then harvested at 2, 6 or 24 h. In some experiments, transcriptional inhibitor actinomycin D (5 μg/ml) was added 30 min before stimulation with TNF and harvested at 6 h. Total cellular RNA was extracted using TriZol method, reverse transcription and real-time RT-PCR were performed as we described earlier [17, 18]. The sequences of primers were as described [19]: PTX3 forward, 5′-GGGACAAGCTCTTCATCATGCT-3′; reverse, 5′-GTCGTCCGTGGCTTGCA-3′; Housekeeping gene glyceraldhyde-3-phosphate dehydrogenase (GAPDH) served as the internal control. Primers for GAPDH are forward primer 5′-AGCAATGCCTCCTGCACCACCAAC-3′ and reverse primer 5′-CCGGAGGGGCCATCCACAGTCT-3′. Total 40 cycles were used and each cycle included denaturation (94 °C, 1 min), annealing (62 °C, 32 s) and extension (72 °C, 1 min 32 s). Real-time quantitative PCR for PTX3 and GAPDH was performed by ABI 7500 Real-Time PCR System and analyzed by 7500 System SDS software version 1.3.1 (Applied Biosystems, Foster City, CA, USA), following manufacturer’s instructions. Product specificity was determined by melting curve analysis and by visualization of PCR products on agarose gels. The amplification of PTX3 gene in stimulated cells was calculated first as the copy number ratio of PTX3 per copy of GAPDH and then expressed as normalized values of fold increase over the value obtained with baseline control cells (time 0).

### PTX3 promoter luciferase reporter constructs and cell transfection

To investigate whether TNF affect PTX3 expression by modulating promoter activity in HASMC, transfection was performed with ExGen 500 according to the manufacturer’s instructions (Fermentas Inc, Mississauga, ON, Canada) using luciferase reporter plasmid harbouring the human PTX3 promoter [20]. Wild type PTX3 promoter spans nucleotides 1-1200 bp (accession number X97748). NF-κΒ and AP1 PTX3 mutated promoter were constructed using site-directed mutagenesis. The vector sequence was confirmed by DNA sequencing. HASMC (4 × 10^4^) were plated into 12-well culture plates in fresh complete DMEM. At 50–70 % confluency, cells were transfected with human PTX3 promoter construct. In each well, 1.6 µg of PTX3 promoter DNA and 0.4 µg of *Renilla* luciferase reporter vector-pRL-TK (Promega) were co-transfected for 24 h. The medium was changed and cells were washed and stimulated with TNF (10 ng/ml) or unstimulated. After 12 h of cytokine stimulation, cells were washed twice with PBS and cell lysates were collected with 100 µl of reporter lysis buffer (Promega, Madison, WI, USA). In some experiments, cells were pretreated for 1 h with U0126 (10 µM), SB203580 (10 µM), and SP600125 (50 nM) or with DMSO before stimulation with TNF (10 ng/ml) for 12 h. The luciferase activity was measured by the Dual-Luciferase Assay System kit (Promega, Madison, WI, USA) using a luminometer (model LB9501; Berthold Bad Wildbad, Germany). Briefly, 20 µl of cell lysate was mixed with 100 µl of Luciferase Assay Reagent II and firefly luciferase activity was first recorded. Then, 100 µl of Stop-and-Glo Reagent was added, and *Renilla* luciferase activity was measured. All values were normalized to *Renilla* luciferase activity and expressed relative to the transfected non-stimulated cells as we described previously [18, 21].

### Statistical analysis

Data obtained from experiments performed in triplicate and repeated at least three times was represented as mean ± SEM. Differences among groups were analyzed using ANOVA together with a post hoc Bonferroni analysis. Non-parametric data were analyzed using the Kruskal–Wallis test followed by the Mann–Whitney U test. P values were considered significant at 0.05 levels.

## Results

### TNF induced PTX3 expression in HASMC via a transcriptional mechanism

**Fig. 1:**
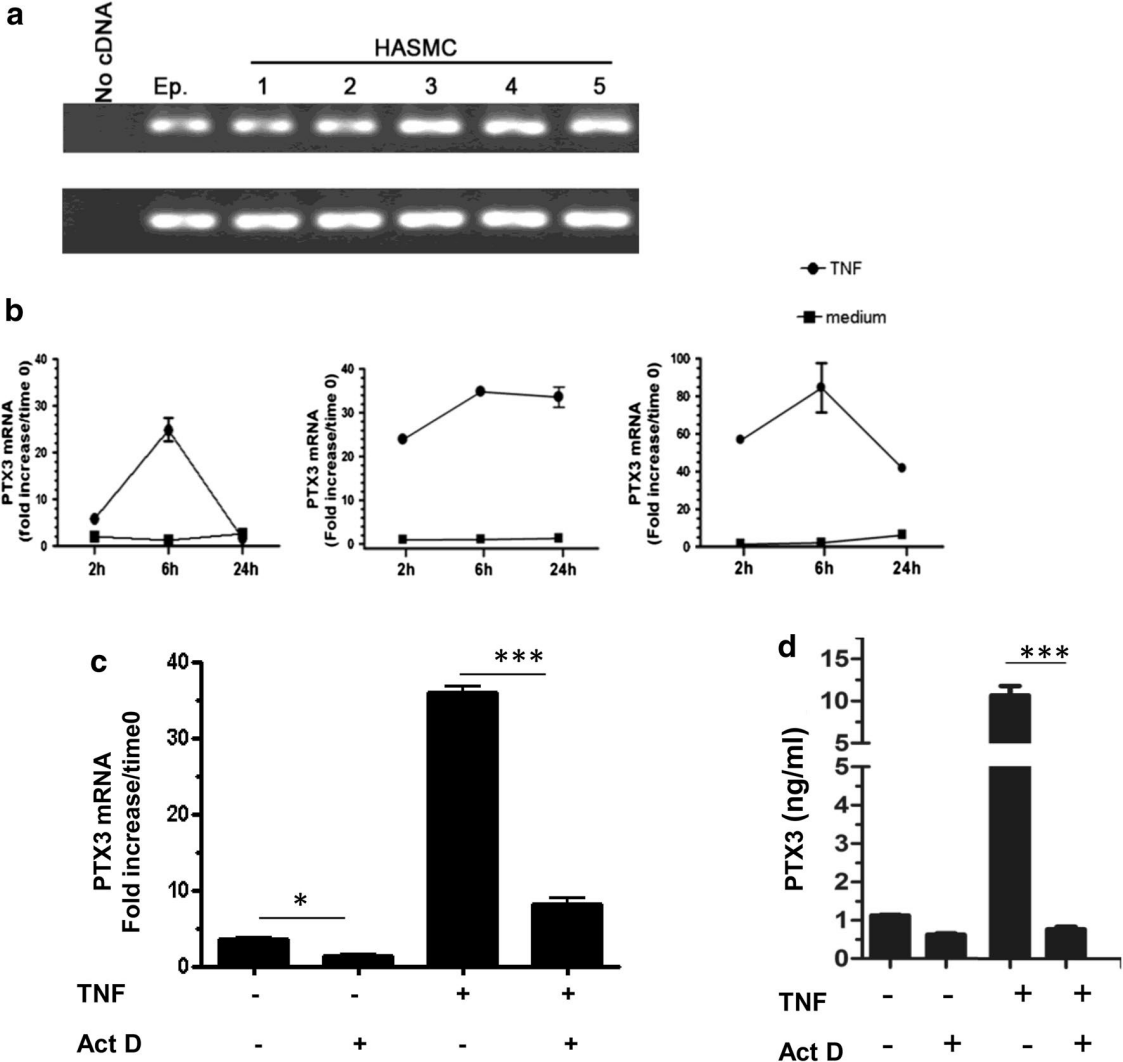
TNF-induced expression PTX3 in HASMC is inhibited by a transcriptional inhibitor. **a** HASMC from five subjects and epithelial cells (Ep) were processed for total RNA extraction. PTX3 mRNA was detected by RT-PCR in HASMCs and human epithelial cells. **b** Serum-deprived HASMC from three donors were stimulated with TNF (10 ng/ml) for 2, 6, and 24 h. Time course effect of TNF (10 ng/ml) on PTX3 mRNA was assessed by quantitative real-time RT-PCR. **c** Cells were pretreated with Act D (5 μg/m) for 30 min before stimulation with TNF and harvested at 6 h. **d** Serum-deprived HASMCs were stimulated with TNF (10 ng/ml), medium or pretreated with Act D for 24 h. PTX3 protein release was assessed by ELISA. ***P < 0.001, *P < 0.05 compared with TNF alone or medium stimulated cells, respectively

We first confirmed in different primary HASMC that TNF stimulation induces PTX3 mRNA expression. RNA preparations from serum-deprived HASMC were first analyzed by RT-PCR. As shown in Fig. 1a, HASMC from five different donors depict constitutive PTX3 mRNA, as observed in primary human epithelial cells (Ep.) used as positive control [19]. Since TNF is one of the critical proinflammatory effector cytokines in asthma, and has been shown to induce multiple inflammatory genes in HASMC [21, 22], we further characterized the kinetic of TNF induced PTX3 mRNA expression using quantitative real-time RT-PCR. HASMC from three different donors treated with TNF showed a significant increase in PTX3 mRNA expression that reached a maximum level at 6 h and tended to decrease at 24 h. TNF induction of PTX3 mRNA expression was variable between the three HASMC tested but showed similar trend (Fig. 1b). Furthermore, in response to TNF (10 ng/ml) stimulation PTX3 protein release by primary HASMCs was time-dependent and reached a maximum at 48–72 h as we previously demonstrated [23] (data not shown).

To investigate whether TNF induces the PTX3 expression is dependent on mRNA neo-synthesis, serum-deprived HASMC were pretreated with the transcriptional inhibitor, Act D (5 μg/ml) and then stimulated with TNF (10 ng/ml) for 6 h. As shown in Fig. 1c, TNF induced PTX3 mRNA in HASMC was significantly reduced upon actinomycin D treatment compared with untreated cells (n = 3, P < 0.001). Similarly, the effect of blocking mRNA neo-synthesis on TNF induced PTX3 protein release by HASMC was also evaluated. As shown in Fig. 1d, pretreatment with Act D before TNF stimulation abrogated the induction of PTX3 protein release in HASMC (TNF: 10.6 ± 2.25 ng/ml compared with 0.76 ± 0.14 ng/ml for TNF + Act D, n = 3, *p* < 0.0001).

**Fig. 2:**
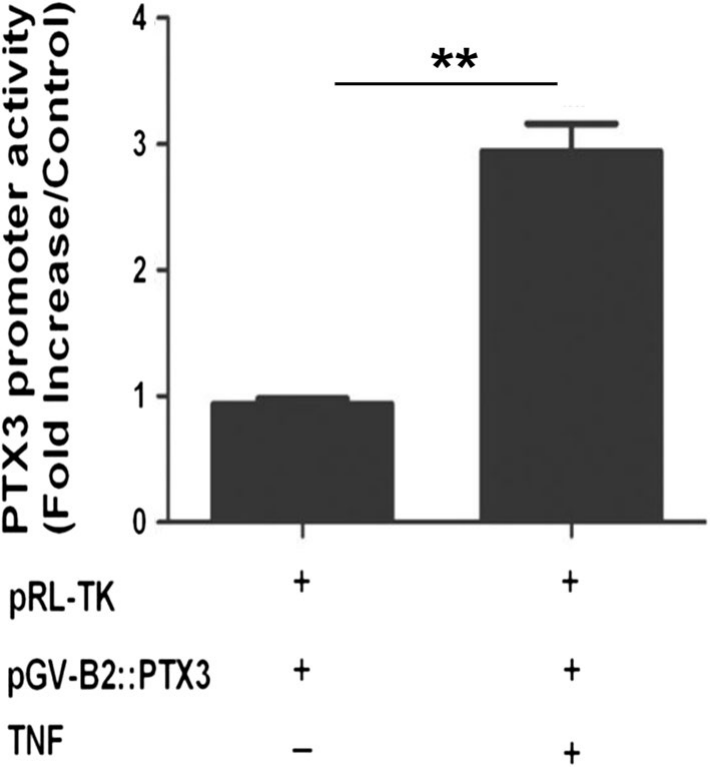
TNF-induced PTX3 promoter activity in HASMC. HASMC were transiently transfected with the PTX3 promoter luciferase construct and stimulated with 10 ng/ml of TNF or vehicle for 12 h. *Values* are presented as the mean fold increase of luciferase activity normalized to the mock renilla luciferase activity from 5 different HASMC (**P < 0.01)

We then determined the effect of TNF on inducing PTX3 promoter activity in transiently transfected primary HASMC. As shown in Fig. 2, HASMC transfected with PTX3 Luciferase promoter construct showed significant increase in luciferase promoter activity in response to TNF (mean value of fold increase of 3.138 ± 0.341 compared with transfected but non-stimulated, n = 5).

### JNK and p42/p44 ERK MAPK pharmacological inhibitors attenuate TNF-induced PTX3 promoter activity in HASMC

**Fig. 3:**
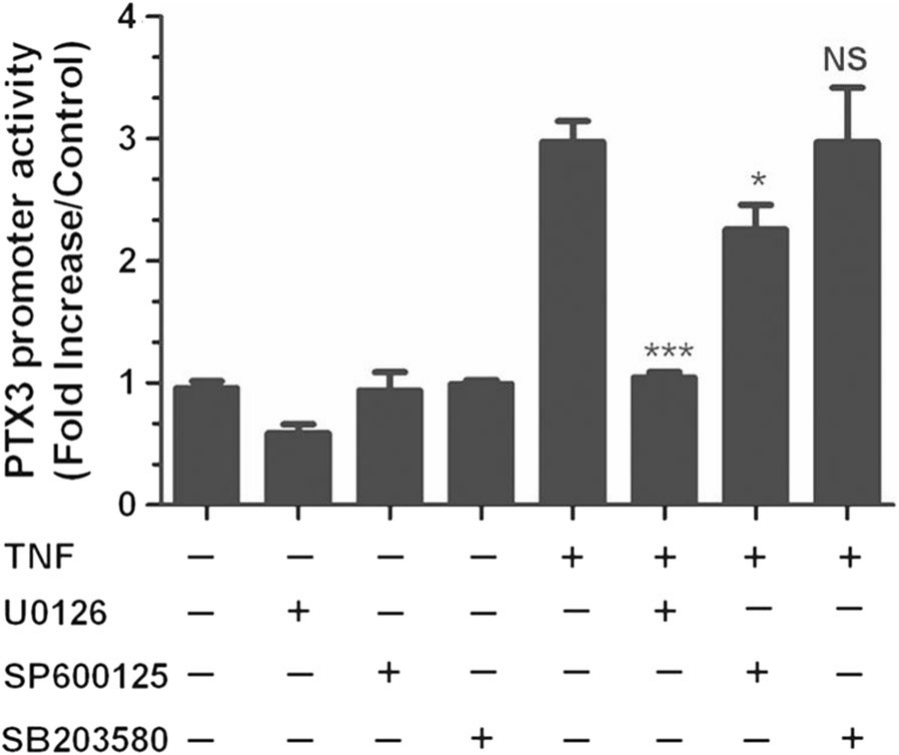
Pharmacologic inhibition of MAPK pathway attenuates the TNF-induced PTX3 promoter activity. Serum-fed HASMC were transfected with luciferase encoding promoter construct of PTX3, followed by stimulation with TNF without or with prior incubation for 1 h with inhibitors of JNK (SP600125, 50 nM), p42/p44 ERK (U-0126, 10 μM) and p38 MAPK (SB-203580, 10 μM), as described in Sect. “Methods”. *P < 0.05, ***P < 0.001 compared with TNF-stimulated alone. *ns* not significant; n = 5

We then investigated whether TNF induction of PTX3 gene expression at the promoter level can be affected by MAPKs inhibitors. The treatment of HASMC with p42/p44 ERK or JNK (U0126 or SP600125, respectively), but not with p38 inhibitors (SB203580) led to a significant inhibition of PTX3 promoter activity (P < 0.001, n = 5) (Fig. 3). No significant effect could be detected on baseline promoter activity in cell incubated with the inhibitors alone (Fig. 3).

### TNF-induced PTX3 protein release is mediated via JNK and p42/p44 ERK MAPK pathways in HASMC

**Fig. 4:**
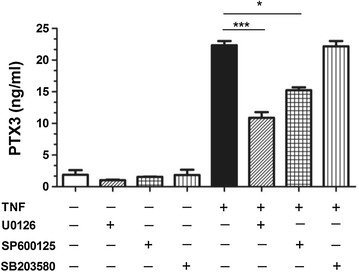
JNK and p42/p44 ERK inhibitors decrease TNF-mediated PTX3 protein expression. PTX3 protein released by HASMC treated with TNF (10 ng/ml) for 24 h with or without pretreatment inhibitors of JNK (SP600125, 50 nM), p42/p44 ERK (U0126, 10 μM) and p38 MAPK (SB203580, 10 μM) was determined by ELISA. *P < 0.05, ***P < 0.001 was compared with TNF-stimulated alone

One of the major downstream pathways for TNF induced cell activation is MAPKs, which play an important role in ASM cell activation for inflammatory response [24]. To characterize the signaling pathways involved in TNF-mediated PTX3 release from HASMC, we performed experiments using pharmacological inhibitors of p38, p42/p44 ERK MAPK and JNK (SB203580, U0126 and SP600125, respectively). Stimulation of HASMCs with TNF increased PTX3 synthesis that was partially blocked by JNK or p42/p44 ERK inhibitors SP600125 or U0126, at 24 h (P < 0.05, n = 5). In contrast, inhibition of p38 with SB203580 has no effect on TNF-mediated PTX3 release by HASMC (Fig. 4). These results indicate that JNK and p42/p44 ERK MAPK are involved in TNF-mediated release of PTX3 by HASMC.

### TNF induced PTX3 promoter activity depends on NF-κΒ and AP1 binding site

**Fig. 5:**
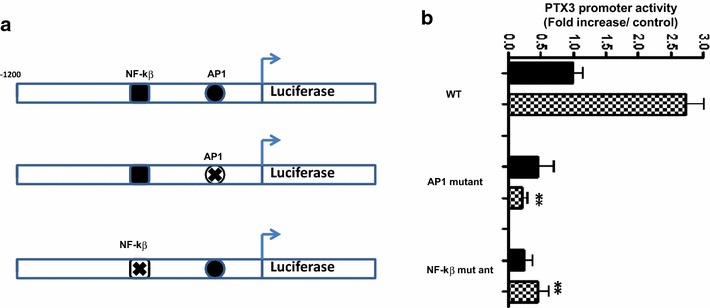
NF-κβ and AP1 mutation abolish TNF induced PTX3 promoter activity. HASMC were transiently transfected with WT PTX3 (−1200 bp), NF-κβ or AP1 mutated reporter luciferase construct (**a**) and stimulated with 10 ng/ml of TNF or vehicle for 12 h. **b** *Values* are presented as the mean fold increase of luciferase activity normalized to the mock renilla luciferase activity from 3 different HASMC (**P < 0.01)

Next, we investigated the importance of response element involved in TNF mediated PTX3 promoter activity in HASMC. We employed reporter plasmids containing PTX3 promoter with mutations in NF-κB or AP1-like transcription factors (Fig. 5a). As observed in Fig. 5b, compared with wild-type (WT) promoter constructs, TNF mediated wild-type PTX3 promoter activity in HASMC was reduced significantly when NF-κB or AP1 binding site were mutated (NF-κΒ mutant: 5.5; AP1 mutant: 11.25 fold decrease compared with WT, n = 3).

## Discussion

PTX3 belongs to the highly conserved superfamily of pentraxins that are characterized by multimeric structure [25]. In comparison with classical short pentraxins such as C-reactive proteins, PTX3 is produced by a variety of cell types upon induction by proinflammatory cytokines. These include among others dendritic cells, neutrophils, macrophages and alveolar epithelial cells [19, 26]. Recently we showed that HASMC are major PTX3 producer in the airways [15, 22]. HASMC constitutively express significant amount of PTX3 that can be enhanced following TNF stimulation. In this study, we showed that TNF induced PTX3 gene expression in primary HASMC occurred via a transcriptional dependent mechanism. Furthermore, PTX3 protein release as well as promoter activity can be inhibited by pharmacological inhibitors of MAPK pathways (JNK and ERK1/2 but not p38). Considering the pleiotropic role of PTX3 in many facets of airway lung biology that encompasses resolution and clearance of inflammatory cells to cellular matrix remodeling these data suggest that within the airway TNF may modulate these events by inducing PTX3. In particular, it is enticing to speculate that TNF induced PTX3 release from HASMC is a feedback mechanism involved in preventing excess of inflammatory response within the airway. In accordance with this possibility, increased tissue damage was observed in PTX3 deficient mice in myocardial infarction model [27], in addition to an enhanced survival of PTX3 transgenic mice upon endotoxemia [28].

TNF is a multifunctional cytokine that is involved in inflammatory reactions in many diseases. Elevated levels of TNF have been detected in sputum, bronchoalveolar lavage (BAL), and biopsy samples from patients with asthma [29]. The central role of TNF in lung inflammation is not only supported by animal models, but also human studies [29, 30]. Previous data shows the anti-TNF therapy as a potential new strategy in severe refractory asthma [22]. In vitro, TNF acts directly on airway smooth muscle to increase contractility, mimicking in vivo hyper-responsiveness [31]. In this regard, our data demonstrating TNF-induced PTX3 transcription and protein release in HASMC suggests a novel pathway by which this cytokine can affect airway structural cells. Whether PTX3 itself affect HASMC contractility needs to be explored. TNF is a strong inducer of eotaxin-1/CCL11 expression in the airway inflammatory [32] and structural cells including HASMC [33] combined with the fact that PTX3 enhanced the eotaxin-1/CCL11 release in HASMC suggests a yet another mechanism whereby TNF induced PTX3 promotes eosinophilic airway inflammation.

Beside its role as proinflammatory cytokine, previous study has showed that TNF inhibits HASMC proliferation via secretion of IFNβ suggesting an important role of this cytokine in regulating airway smooth muscle remodeling. We previously showed that PTX3 inhibits FGF2 induced cell migration, an important factor contributing to increase smooth muscle mass in asthma [15]. This data combined with enhanced expression of PTX3 in smooth muscle bundle within bronchial biopsies of severe asthmatics [15] suggests that TNF mediated PTX3 expression by HASMC may play a protective role in the airways. In agreement with possibility, recent studies have demonstrated that the absence of PTX3 leads to tissue damage exacerbation in mouse model of skin inflammation [13] and that PTX3 derived mesenchymal stromal cells played an essential role in wound repair [14].

TNF exerts its pleiotropic actions on ASMC by binding primarily to p55 or TNFR1 leading to cytokines and chemokines release [34], agonist-induced calcium signals, cell proliferation, and expression of adhesion molecule ICAM-1 [35]. Furthermore, TNF is a well known critical factor modulating mitogen-activated protein kinases (MAPKs) activation in HASMC [36]. In this study, we sought to investigate the signal transduction pathways regulating TNF induced PTX3 promoter activity. Our results demonstrate a critical function for Erk-1/2 since inhibition of upstream MEK-1/2 kinase with U0126 abrogated PTX3 promoter activity mediated by TNF compared to the respective controls. Interestingly, ERK1/2 inhibitor only partially reduced promoter whereas p38 MAP inhibitor has no effect. Furthermore, mutation of NF-κβ or AP1 inhibits significantly TNF induced PTX3 promoter activity in HASM. Our data are in concert with the role of MAPK in mediating transcriptional regulation of NF-κB and AP-1. In fact, TNF induced MAPK activation can mediate the nuclear translocation and enhancement of the transcriptional activity of NF-κB and c-Jun later being known as AP-1, once heterodimerize with c-fos [37].

Pharmacological inhibitors of MAPK (JNK and ERK1/2) significantly inhibited the TNF-induced PTX3 production in HASMC. The effect of JNK on PTX3 protein expression is consistent with prior observations in human lung epithelial cell line [19]. In contrast, PTX3 protein release following p38MAPK inhibition was not affected. Taken together, these data suggest the involvement of other regulatory mechanisms associated with PTX3 expression.Therefore, further studies are required to understand the mechanisms governing PTX3 expression in HASMC, which may include multiple kinases, and transcription factors.

In conclusion, our data provide the evidence that TNF induced PTX3 expression in HASMC is mediated via MAPK (JNK and ERK1/2) as well as AP1 and NF-κβ activation.

## Abbreviations

HASMC: Human airway smooth muscle cells
PTX3: Pentraxin-3
ERK: Extracellular signal-regulated kinase
MAPK: Mitogen-activated protein kinase
JNK: c-Jun N-terminal kinase
BAL: Bronchoalveolar lavage
Act D: Actinomycin D
